# Virtual Grasping: Closed-Loop Force Control Using Electrotactile Feedback

**DOI:** 10.1155/2014/120357

**Published:** 2014-01-02

**Authors:** Nikola Jorgovanovic, Strahinja Dosen, Damir J. Djozic, Goran Krajoski, Dario Farina

**Affiliations:** ^1^Department for Systems, Signals and Control, Faculty of Technical Sciences, University of Novi Sad, Novi Sad, Serbia; ^2^Department of Neurorehabilitation Engineering, University Medical Center Göttingen, Georg-August University, Göttingen, Germany

## Abstract

Closing the control loop by providing somatosensory feedback to the user of a prosthesis is a well-known, long standing challenge in the field of prosthetics. Various approaches have been investigated for feedback restoration, ranging from direct neural stimulation to noninvasive sensory substitution methods. Although there are many studies presenting closed-loop systems, only a few of them objectively evaluated the closed-loop performance, mostly using vibrotactile stimulation. Importantly, the conclusions about the utility of the feedback were partly contradictory. The goal of the current study was to systematically investigate the capability of human subjects to control grasping force in closed loop using electrotactile feedback. We have developed a realistic experimental setup for virtual grasping, which operated in real time, included a set of real life objects, as well as a graphical and dynamical model of the prosthesis. We have used the setup to test 10 healthy, able bodied subjects to investigate the role of training, feedback and feedforward control, robustness of the closed loop, and the ability of the human subjects to generalize the control to previously “unseen” objects. Overall, the outcomes of this study are very optimistic with regard to the benefits of feedback and reveal various, practically relevant, aspects of closed-loop control.

## 1. Introduction

Human grasping is characterized by a remarkable flexibility. Humans can easily grasp, lift, and manipulate objects of very different properties (e.g., texture, weight, and stiffness). Obviously, this process requires an advanced control of grasping forces, which is in human motor control implemented through a blend of feedforward and feedback mechanisms [1]. The former is well reflected in the paradigm of economical grasping: humans use previous sensory-motor experience to scale appropriately the grasping forces according to the expected (estimated) weight of the target object. The goal is to minimize the forces and thereby energy expenditure, and yet avoid slipping. However, this specific mechanism and also grasping as a whole can be significantly impaired when somatosensory feedback pathways are not fully functional due to a disease of the nervous system (e.g., multiple sclerosis [2], deafferented patients [3]).

After an amputation of the hand, a prosthetic device can be used as a functional and morphological replacement of the lost limb. To control the artificial limb, the intention of the user can be inferred from the recorded activity of the user's muscles (myoelectric control). This method, which essentially implements the feedforward pathway between the brain and artificial limb, has been in routine use in commercially available prostheses for decades [4, 5]. However, none of the commercial systems provides any deliberate somatosensory feedback to the user to close the control loop.

Providing somatosensory feedback is a well-known, long standing challenge in the field of prosthetics. The researchers have been investigating various approaches to provide feedback artificially, ranging from direct neural stimulation [6] to noninvasive sensory substitution methods [7, 8]. In the latter, the state of the prosthesis (e.g., joint angles or grasping forces) is communicated to the user by stimulating the skin of the residual limb using mechanical (e.g., vibration motors [9–11], pressure cuffs [12], and motor driven pushers [13]) or electrical stimulation [14]. Closed-loop control of grasping force was most commonly tested. This is not surprising since an appropriate grasping force is necessary for safe lifting and object handling and since this variable cannot be assessed directly using vision (contrary to, for example, hand joint angles).

Although there are many studies presenting closed-loop systems, only a few of them objectively evaluated the closed-loop performance, mostly using vibrotactile stimulation. The conclusions of earlier studies are however partly contradictory. Some found that the feedback improved the performance [12, 13, 15], while others did not find a clear advantage of the closed-loop control [11]. In some studies, the feedback was beneficial only under certain conditions (e.g., feedforward uncertainty [16] and experienced subjects [17]).

In the case of electrical stimulation, systematic evaluation is very scarce. Scott et al. [18] reported subject satisfaction with the provided electrotactile feedback while Wang et al. [19] stated that the users could differentiate appropriate gripping force for a wide variety of different activities, but in both cases the claims were not backed up with the actual results. Lundborg et al. [20] evaluated two-channel electrical stimulation feedback in four patients with sensory impairments (recent median nerve repair) and a single user of a myoelectric prosthesis. In all subjects, the performance in force control during a force matching task was better when using electrical stimulation with respect to the condition with no tactile feedback. Only one level of force, selected as comfortable by the subject before the test, was used during the task. Zafar and Van Doren [21] tested a single channel electrical stimulation feedback using a specialized setup simulating grasping of a compliant object, thereby allowing the subject to exploit visual cues for the force control in parallel to the tactile feedback. It was found that the supplemental feedback slightly improved the force control even when additional visual cues were provided.

The goal of the current study was to systematically and objectively investigate the capability of human subjects to control grasping force in closed loop using electrotactile feedback. We have developed a virtual setup that allowed us to investigate different aspects of the closed-loop control. The setup is also realistic in the sense that it operates in real time and includes a set of real life objects, as well as a graphical and dynamical model of the prosthesis. We have used the setup to investigate the role of training, feedback and feedforward control, robustness of the closed loop, and the ability of the human subjects to generalize the control to previously “unseen” objects.

## 2. Methods

### 2.1. Subjects

The experiment was carried out in the Biomedical Engineering Lab at the Department for Systems, Signals and Control, Faculty of Technical Sciences, University of Novi Sad, Serbia. Ten healthy, able bodied volunteer subjects (5 males and 5 females, 28 ± 3 years old) participated in the experiment after signing the consent form which was approved by the local ethical committee.

### 2.2. Experimental Setup

**Figure 1:**
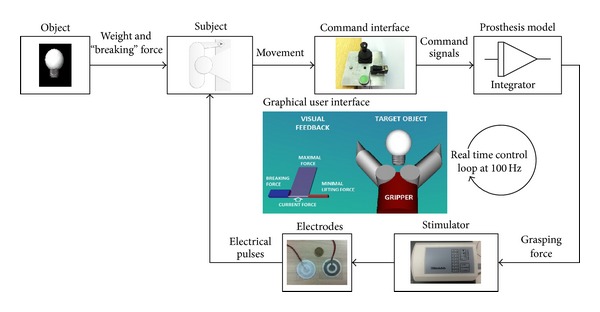
Experimental setup for closed-loop force control using electrotactile stimulation. The task for the subject was to generate an appropriate force to lift the target object without breaking it. The command interface was joystick and a push button. The electrotactile feedback was provided using a stimulation unit and concentric electrodes placed on the forearm of the subject. The control loop operated in real time at the sampling frequency of 100 Hz. The graphical interface for the subject included a model of the prosthesis, target object, and a force bar showing the minimal, current, maximal and breaking force. The bar plot was used at the beginning of the training to teach the subjects the task they should perform as well as the meaning of the electrotactile feedback. Otherwise, the visual force feedback was not shown on the screen. For detailed explanation see Section 2.4.

The experimental setup (see Figure 1) comprised the following components: (1) single-axis contactless joystick (CH Products, USA), (2) mechanical pushbutton, (3) current-controlled multichannel stimulator TremUNA (UNA systems, SRB), and (4) a standard desktop computer (host PC) equipped with a data acquisition card (PCI 6024, National Instruments, USA). The control program for virtual grasping experiments was implemented using Matlab 2012b and Simulink, Simulink 3D Animation, and Real Time Windows Target toolboxes. The signals from the joystick and the mechanical pushbutton were acquired by the DAQ card and supplied as the control inputs to the model of the prosthesis. The control loop operated in real time at the sampling frequency of 100 Hz. Based on the grasping force generated by the prosthesis, the stimulation unit provided the electrotactile feedback to the user. The unit included eight stimulation channels in total but only two were actually used in the current experiment. The stimulation parameters were controlled in real time from the host PC via a USB port, and the current pulses were delivered by using self-adhesive concentric electrodes (CoDe 501500, 4 cm diameter, SpesMedica, IT).

The graphical user interface depicted a model of a simple, single degree of freedom prosthetic hand (gripper) and the object that was the target for grasping. The object was positioned between the fingers of the prosthesis so that when the hand closed, it grasped the object. In some experimental conditions, visual force feedback was also provided to the subject in the form of a bar graph (see Figure 1 and Section 2.5). The virtual hand was controlled by the joystick and it operated as a first order, velocity-controlled system (i.e., transfer function *G*(*s*) = 0.5/*s*). Before the hand contacted the object, the joystick inclination was proportional to the speed of hand closing, whereas after the contact has been made, the joystick inclination was proportional to the buildup rate of the grasping force. This was similar to the “gated ramp controller” that was also used in [16, 22]. The pushbutton indicated the end of force control phase and the start of the object lift-off phase (see Section 2.4).

Joystick was selected since it provided a stable feedforward interface. Using myoelectric control would have been possible as well; however, in the current study the focus was on the feedback interface and therefore the influence of the other system components was minimized by assuming ideal behaviors. The gated ramp controller was adopted since it is similar to the control method actually used in one of the commercially available hands (i-Limb from Touch Bionics [4]) and also since this approach effectively “decoupled” the position of the joystick from the amplitude of the grasping force; if the force would be controlled proportionally as in some other prosthesis (Sensor Hand Speed and Michelangelo Hand from Otto Bock [5]), the subjects could easily estimate the current force level directly from the joystick inclination and without using electrotactile feedback information. In addition to the possibility to “safely” conduct tests which could be difficult to realize in real life (i.e., breaking objects), an advantage of the virtual setup was that it provided very controlled feedback and prevented incidental sources of information which would be available if the users controlled a real prosthesis (e.g., motor sound and vibrations).

### 2.3. Electrotactile Feedback

Electrotactile feedback was provided using two bipolar concentric electrodes placed on the dorsal side of the subject forearm, approximately midway between the elbow and wrist. The stimulation was delivered in monopolar configuration: the inner fields were used as the cathodes and the outer rings were connected together as a common anode. The stimulation was monophasic compensated; rectangular depolarizing pulse injecting charge to activate cutaneous afferents and elicit tactile sensation was followed by an exponential discharge waveform of opposite polarity to remove the charge out of the tissue. The rate of pulse delivery was constant and set to 100 Hz. This frequency was selected since pilot tests demonstrated that it elicited a well-localized, continuous sensation (i.e., responses to individual pulses fused together) resembling constant pressure on the surface of the skin. The current amplitude was also constant and set to 3 mA, and the pulse width was modulated within the dynamic range of the stimulation. The latter was determined for each subject individually as the interval between 1.2∗ST and 0.8∗PT, where ST and PT are sensation and pain thresholds tested before the start of the experiment. The thresholds were detected using the method of limits [23]; that is, the pulse width was set to minimal value (50 *μ*s) and then successively increased in steps of 50 *μ*s until the subject indicated that he/she felt the stimulation (SP) or that the stimulation became painful (PT). This procedure was repeated three times in succession, and the average was used as the final value. The scaling factors for ST and PT defining the dynamic range [1.2∗ST, 0.8∗PT] were adopted heuristically to assure that the minimal stimulation within the dynamic range can be perceived by the subject (pulse width > ST) while maximal stimulation is still nonpainful (pulse width < PT). The stimulation was proportional to the force; that is, normalized force (0-1) was linearly mapped to the dynamic range. Stimulation intensity was modulated by adjusting the pulse width since this allowed a finer control of elicited sensations compared to changing the current intensity (pilot tests). The two electrodes delivered the same information, that is, the same force scaled between the respective electrode thresholds as explained above. This configuration was selected since the pilot tests demonstrated that two electrodes provided better quality and discriminability of sensation compared to the use of a single electrode.

### 2.4. Virtual Grasping Task

At the beginning of the trial, a graphical model of the object that was the target for grasping was shown, positioned directly in front of the gripper and in-between the fingers, as explained before. Each object was characterized by two parameters: minimal grasping force needed to successfully lift the object and maximal allowed grasping force (“breaking” threshold). In total, 19 different objects were used for the experiment (see Table 1). The limits for each object (min/max force in Table 1) are expressed as normalized force, where 1 corresponds to the maximal force that the virtual prosthesis could produce. The force limits were selected heuristically but the goal was to reflect the reality as much as possible. The minimal force was proportional to the weight of the object, and the maximal force was set to about 130% of the minimal force. The role of the maximal force was to prohibit the subjects from using excessively high forces to lift the objects, enforcing the paradigm of economical grasping (i.e., minimizing grasp forces while avoiding object slip [16]). Without this constraint, each object could have been securely grasped simply by generating the maximum force. However, generating too high grasping forces during the real life not only would mean excessive energy expenditure and lower battery life (i.e., noneconomical prosthesis use) but could also damage or even break the objects (e.g., a light bulb or an egg). Note that it was not important for the force limits to strictly reflect the reality, since the subjects anyway got the opportunity to “learn” the objects before the performance was evaluated (see Section 2.5). The goal was to give more general, absolute (e.g., heavy or light) or relative (i.e., heavier/lighter than the previous object), visual cues about the object weight.

**Figure 2:**
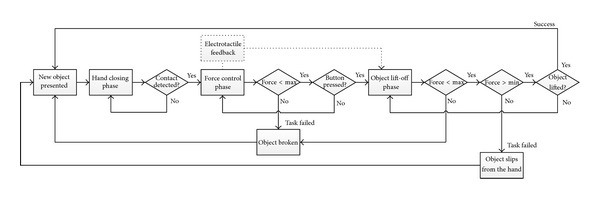
The steps comprising a single trial of a virtual grasping. There were three phases: (1) hand closing, (2) force control, and (3) object lift-off. The trial was deemed successful if the object was successfully lifted, while keeping the grasping force within the predefined limits (target window) for the given object.

**Figure 3:**
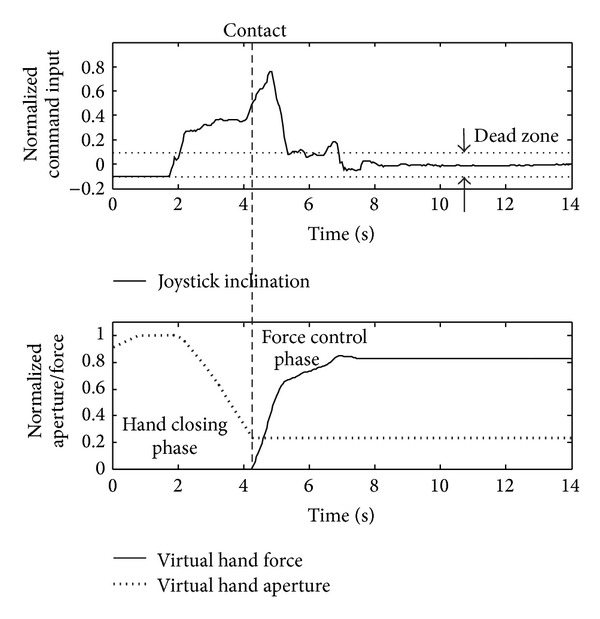
Control signals and prosthesis state variables recorded during a representative trial. In the hand closing phase, the joystick controls the hand aperture and after contacting the object, it controls the grasping force. Initially the subject increased the force faster to bring the signal in the vicinity of the target window. Afterwards, the subject becomes more careful, slowing down the force increase and performing fine corrections. To achieve stable control, a dead zone for the joystick signal was adopted.

The task for the subject was to grasp and lift the object by generating force that was within the predefined limits (target window), that is, enough to lift the object and yet lower than the “breaking” force. The hand was initially fully opened. The subject started hand closing by pushing on the joystick. After the contact was detected, the joystick controlled the grasping force, as explained before. At the same time, the electrotactile feedback was activated. All the objects were absolutely stiff (no deformation, i.e., static grasp) and therefore the visual cues about the developed force were not available. When the subject judged that the grip strength was appropriate, he/she pressed the button to signal that the hand should lift the object. If the force was above the lower limit, the hand would successfully lift the object (task successfully accomplished). Otherwise, if the force became less than the lower limit anytime during the object lift-off, the object would slip from the hand (task failed). Similarly, if the grasping force went above the higher limit for the given object anytime during the trial, the gripper would immediately “fall through,” signaling that the object has been broken (task failed). The steps within the trial are shown in Figure 2, while Figure 3 depicts the relevant signals and prosthesis behavior.

### 2.5. Experimental Protocol

The subjects were comfortably seated in a chair in front of the table so that he/she was able to operate the joystick and a pushbutton. The experimental session comprised training and evaluation, and each phase included several conditions which are described in sequel. In total, the experiment lasted approximately 2.5 h. Before starting, the experiment was explained to the subject, the stimulation thresholds were determined, and the subject was allowed to practice virtual grasping with one object for approximately 5 min (simultaneous electrotactile and visual feedback). The goal was for the subject to familiarize with the setup and the task, to accommodate to the sensation of continuous stimulation, and also to learn the force coding through electrotactile stimulation.

The training phase comprised the following conditions.

*(i) TR-VIS-ELE: Training with Visual and Electrotactile Feedback.* The subject performed virtual grasping trials with five different target objects, while visual (force bar) and electrotactile force feedback were simultaneously provided. Due to visual feedback (see Figure 1), this was an easy task and each object was grasped two times. The objects were selected to span the full range of normalized weights (0-1), and they were ordered and presented to the subjects according to their weight, from light to heavy (i.e., see Table 1, objects 1, 2, 3, 4, and 11). The goal of this step was for the subjects to become familiar with a set of objects by learning their weights and corresponding electrotactile sensations assisted by the full visual feedback about force (force bar). 

*(ii) TR-ELE-1: Training with Electrotactile Feedback and Known Objects.* The same five objects as in TR-VIS-ELE were presented again and in the same order, but this time only the electrotactile force feedback was given. Each object was presented repeatedly, until the subject accomplished the grasping task successfully two times in a row or until the maximum number of trials was exceeded (15 trials). After grasping and lifting the object two times in succession, we assumed that the subject learned to adjust the correct force for that particular object by relying on the electrotactile feedback.

*(iii) TR-ELE-2: Training with Electrotactile Feedback and “Unseen” Objects.* The procedure was the same as in TR-ELE-1 but this time a new set of five objects was used. Again, the objects were selected to sample the full range of weights, and they were presented to the subjects ordered according to their weights (i.e., see Table 1, objects 7, 8, 10, 5, and 6). This condition was compared to the previous one. The goal was to evaluate if the subjects could generalize the principles learned in TR-VIS-ELE and TR-ELE-1 to grasp a set of novel objects in this condition using only electrotactile feedback (i.e., without previously revealing the object weight through simultaneous electrotactile and visual feedback).

The evaluation phase comprised the following conditions.

*(i) TE-FDB: Closed-Loop Performance Test.* Ten objects used in TR-ELE-1 and TR-ELE-2 were presented to the subject, each object twice in succession (20 trials in total). The goal of this test was to determine the baseline closed-loop control performance, that is, the performance of grasping ten objects which were used during the training. This was the control condition for all the other tests to follow. 

*(ii) TE-FWD: Feedforward Test (No Feedback).* The procedure was the same as in the previous test, but this time no force feedback was provided. This condition was compared to TE-FDB to test to what extent the subjects relied on the feedback for the task accomplishment. Since the prosthesis was modeled as an ideal integrator, the grasping task could be accomplished without using the feedback. Instead, the subjects could set the joystick in a certain position and count the time needed for the force to increase to a desired value (pure feedforward control). To further simplify the task for the subjects, the hand was already in contact with the object at the beginning of the trials in this condition (i.e., no need to close the hand and visually confirm that the contact has been made). 

*(iii) TE-FWD-ALT: Feedforward Test (No Feedback) with Altered System Parameters.* The procedure was as in the previous condition, but the prosthesis model *G*(*s*) was changed (i.e., integrator gain doubled, *G*(*s*) = 1/*s*), making the force responding two times faster to the joystick command. The goal was to test how robust was the feedforward control to the change of the model parameters. Since there were two trials per object and the set of objects was known, the assumption was that the subjects could implicitly discover that the system behavior has been changed, for example, by using the task accomplishment or failure as the feedback to update the control in the subsequent trials.

*(iv) TE-FDB-ALT: Closed-Loop Performance Test with Altered System Parameters.* The procedure was the same as in the previous condition, but with the electrotactile feedback provided. If the subject used the feedback with the original system, this test would show how robust closed-loop control was with respect to a significant change in the system behavior. If the subjects used feedforward control with the original system, this test would show if closing the loop was useful at least when there was a change in the system behavior (uncertainty).

*(v) TE-FDB-GEN: Closed-Loop Performance Test for Generalization.* In this test, objects were presented in pairs (see Table 2). First, one object used in the previous conditions was presented as a reference, and the grasping trials were repeated with the same object until the task was accomplished successfully. Then, a novel, test object was presented, where this object was “derived” from the reference in several ways: (1) similar weight as the reference, (2) a composite comprising a stack of the reference objects (e.g., two reference objects packed together), and (3) scaled version of the reference (i.e., scaled up or down in volume with respect to the reference). The first case was essentially a classical force matching task [17] while in the two other cases the subjects had to accomplish a “multiplication”/“division” in the space of force/electrotactile sensations. The goal of this step was to test the ability of the subject to solve the type of tasks that we envision the users of the prosthesis could face during the real life application of the device (i.e., grasping a novel object that can be related through the user's experience to a similar object that was handled in the past).

Contrary to training conditions in which the objects were presented ordered according to their weight (from lighter to heavier), during all the evaluations the objects were presented in the random order.

### 2.6. Data Analysis

The performance was measured using the following outcome measures.

- *Average number of attempts (ANA):* This performance index was used to evaluate the training and it was defined as the average number of grasping trials per object before the subject “learned” to grasp the object, that is, before the object was grasped successfully two times in succession.
- *Success rate in task accomplishment (SR):* As explained before, if the subject successfully lifted the object without object slipping or breaking, the trial was deemed successful. Success rate was expressed in percent.
- *Force error (FE):* FE was calculated as the difference between the minimal force to lift the given object and the applied grasping force, but only considering those trials in which the grasping force was not high enough and the object thereby slipped from the grasp. FE evaluated the average level of undershooting and it was adopted as a more sensitive, continuous measure of performance compared to SR, which had only a binary outcome (success or failure).
- *Time to accomplish the task (TAT):* This was the time from the beginning and until the end of the trial (success, slip, and break).

Data analysis was performed using custom functions written in MATLAB 2012b (MathWorks, US). Statistical tests were performed using STATISTICA 10 (StatSoft, US). Repeated measures ANOVA with the experimental condition as the within-subject factor was used for the group comparison and Tukey's honestly significant difference criterion for the post hoc pairwise tests. The data were tested for sphericity (Mauchly's sphericity test). The threshold for the statistical significance was set to *P* < 0.05.

## 3. Results

**Figure 4:**
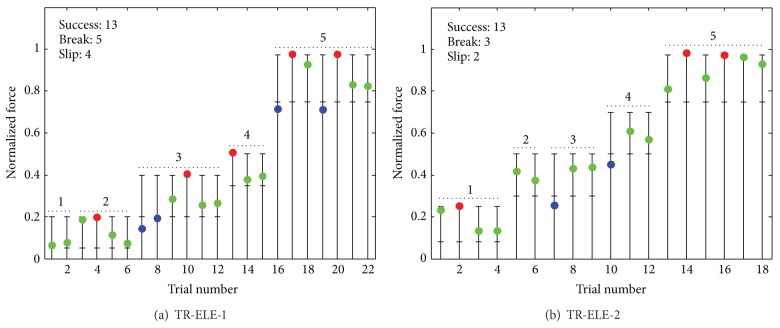
Representative results of the training: (a) TR-ELE-1 and (b) TR-ELE-2. The horizontal lines are force limits, green circle is a successful grasp and lift, blue circle denotes the trial in which the object slipped from the grasp (i.e., grasping force lower than the minimal necessary force), and the red circle represents the trials in which the object was broken (i.e., grasping force crossed the upper limit). The trials with the same object are grouped by a dashed line. From trial to trial, the subject adjusted the grasping force, eventually reaching the target force window. The training was faster in TR-ELE-2.

**Figure 5:**
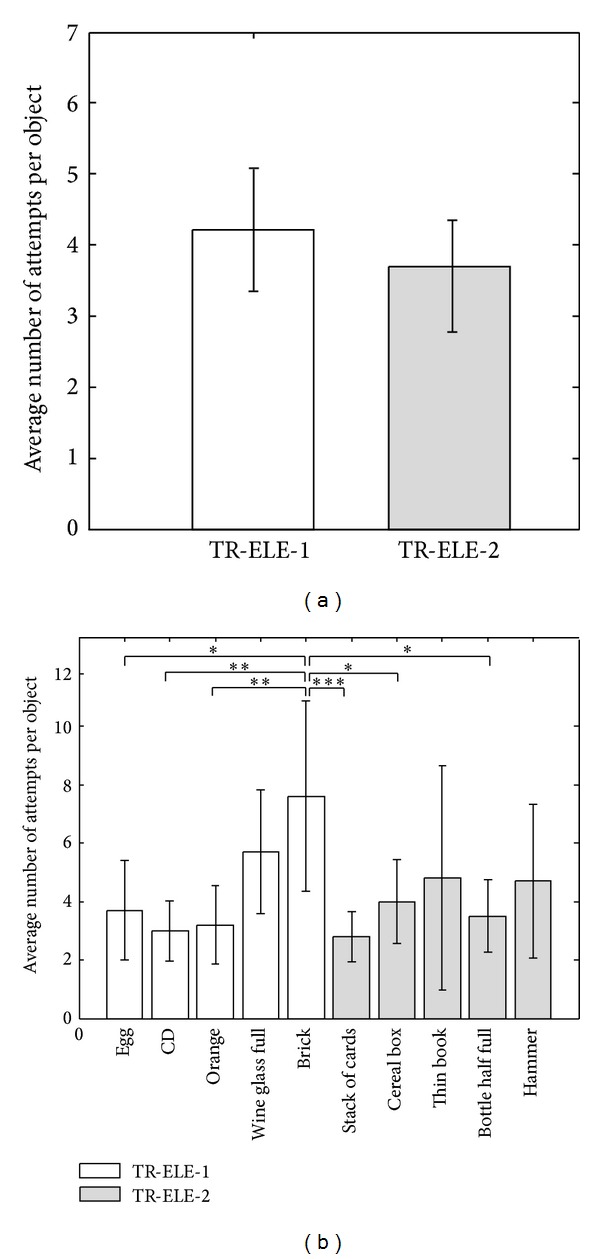
Overall training results (i.e., average number of attempts per object ± standard deviation): (a) across conditions and (b) across objects. The training took similar number of trials in TR-ELE-2 (“unseen” objects) and TR-ELE-1 (previously “seen” objects). In addition, in TR-ELE-2 the subjects learned how to handle heavy objects, which were particularly challenging in TR-ELE-1. Within each condition, the objects in (b) are arranged by their weight (*P < 0.05; **P < 0.01; ***P < 0.001).

The average sensation and pain thresholds were 120 ± 50 *μ*s and 500 ± 50 *μ*s, respectively.  Figure 4 shows a representative result from the second and third training condition (TR-ELE-1 and TR-ELE-2). It can be seen (Figure 4(a), third object) that the subject was adjusting the force in the next trial based on the outcome of the previous one, reaching the target window in a few steps. In the case of light and medium objects (e.g., object 3 in TR-ELE-1), these steps were rather small, suggesting fine control, whereas for the heavy object (e.g., object 5 in TR-ELE-1), the adjustments were more crude, producing trial by trial oscillations above and below the target window. Fewer trials were needed to complete the training in TR-ELE-2. The summary results of the training are given in Figure 5. The average number of attempts (ANA) per object was similar in TR-ELE-2 and TR-ELE-1 condition (Figure 5(a)), despite the fact that in TR-ELE-2 the subjects faced a set of objects that were not handled before using visual force feedback (as in TR-VIS-ELE and TR-ELE-1). In addition, it seems that heavier objects posed a challenge for the subjects in TR-ELE-1, while in TR-ELE-2 the performance was similar across all objects (i.e., no statistically significant differences between light and heavy objects). The data for individual objects (Figure 5(b)) did not pass the sphericity test, and therefore ANOVA with Greenhouse-Geisser correction was used in this case, and it showed that there was a significant difference between the objects (adjusted *P* < 0.05).

**Figure 6:**
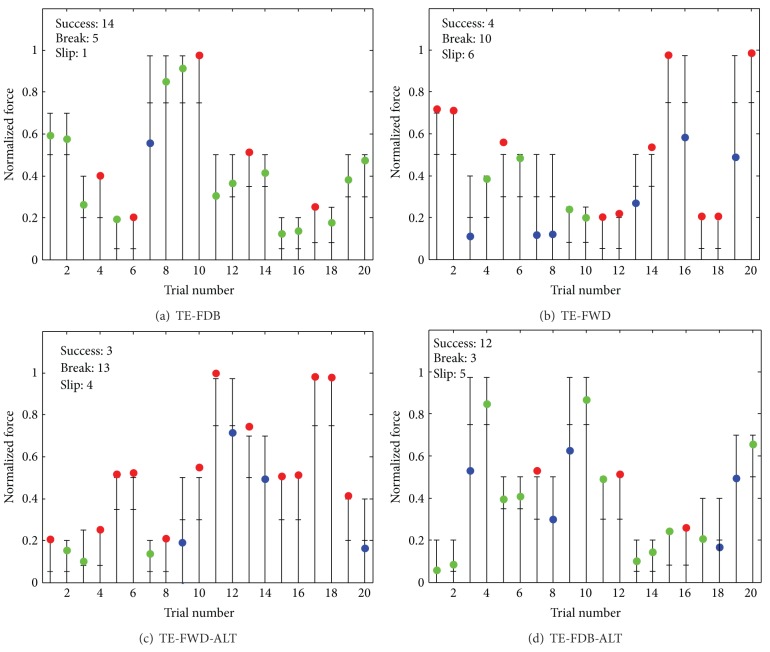
Representative results for the testing conditions: (a) TE-FDB, (b) TE-FWD, (c) TE-FWD-ALT, and (d) TE-FDB-ALT. The horizontal lines are force limits, green circle is a successful grasp and lift, blue circle denotes the trial in which the object has slipped from the grasp (i.e., grasping force lower than the minimal necessary force), and the red circle represents the trials in which the object was broken (i.e., grasping force crossed the upper limit). For each of the ten objects, there were two grasping trials in succession. The performance was much better when the feedback was provided.

Figure 6 illustrates the results from the four testing conditions. For this particular subject, the success rate during the closed-loop control was 70% (TE-FDB) and the performance was similar even after the system behavior was significantly changed (SR of 60% in TE-FDB-ALT). Without feedback, the performance was low (20% in TE-FWD and 15% in TE-FWD-ALT). In the conditions with feedback, the subject was more successful in hitting the target force window already in the first trial (i.e., 7 out of 10 in TE-FDB versus 1 out of 10 in TE-FWD). Furthermore, if the first trial was unsuccessful, the subject was better in correcting the mistake in the second trial. Without feedback, the subject tried to correct as well, but he/she was not very precise, often first overshooting (break) and then in the very next trial undershooting (slip) or vice versa (e.g., see trials 13-14 and 15-16 in TE-FWD; Figure 6).

**Figure 7:**
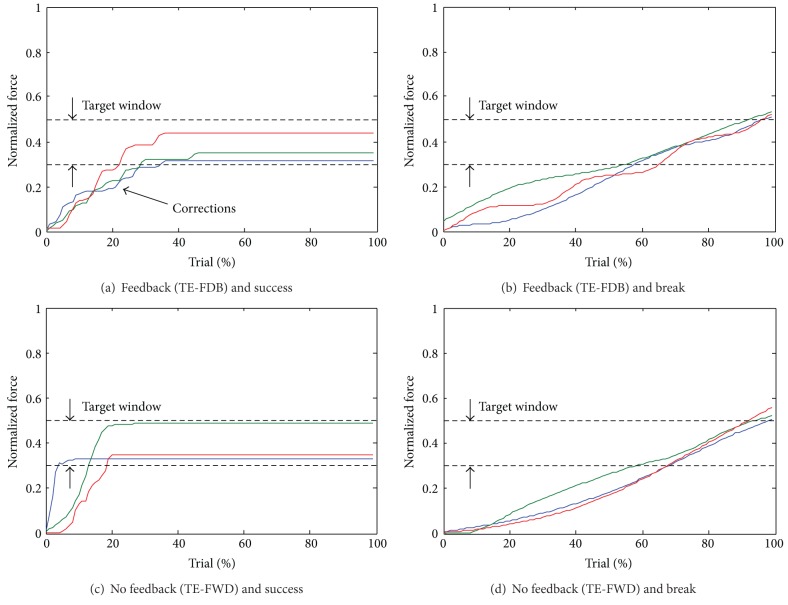
Representative examples of forces generated by different subjects (color profiles) grasping the same object in different testing conditions: (a) and (b) for successful grasping and breaking during TE-FDB, respectively, and (c) and (d) for successful grasping and breaking during TE-FWD, respectively. In both conditions, the subjects would steadily increase the force, but when the feedback was provided, they would also adjust the rate of force increase many times during the trial (e.g., see corrections in (a)). The time is normalized to the duration of the trial in order to emphasize the similarity in the shape of the force profiles for different subjects in the same condition.

The characteristics of the human control in different conditions can be seen from the force traces depicted in Figure 7. Different subjects exhibited similar control strategies in the same condition. When the subjects were provided with electrotactile feedback, they would steadily increase the force, but they would also modulate the rate of force increase many times during the trial (several joystick adjustments). Without feedback, the subjects would simply increase the force at a constant rate by always keeping the joystick at one selected inclination.

**Figure 8:**
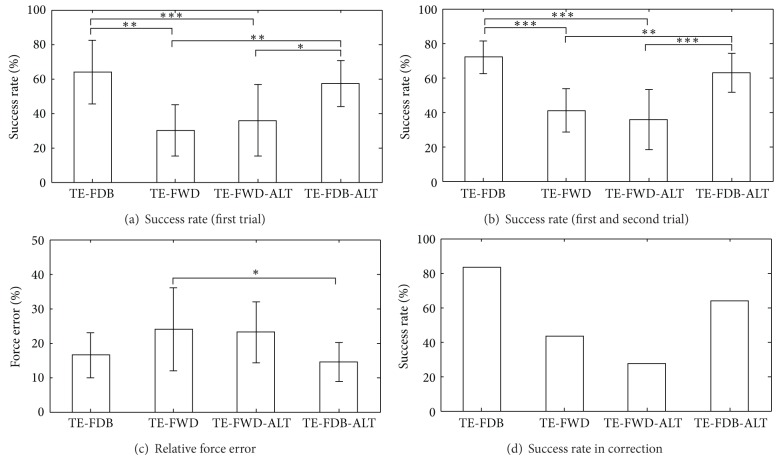
Overall results (mean ± standard deviation) in different testing conditions: (a) success rates (SR) from the first trials only, (b) SR from the first and second (correction) trials together, (c) force errors (FE) in the trials in which the object slipped from the grasp, and (d) SR in corrections (second trial was successful after the first had failed). In (d), only a grand average is reported, since the number of failed first trials was very different between subjects and conditions (30/36, 29/67, 12/44, and 28/44 for overall corrected/failed, left to right). The subjects were more successful in grasping the objects, made smaller errors in the failed trials, and better corrected the unsuccessful trials when the feedback was provided (*P < 0.05; **P < 0.01; ***P < 0.001).

The overall results from the testing phase are given in Figure 8. The average performance during closed-loop control (Figures 8(a) and 8(b)) was 64 ± 18% for first trials only and 72 ± 10% for first and second (correction) trials together. During feedforward control, the success rate dropped significantly: 30 ± 15% and 41 ± 13% in TE-FWD and 36 ± 21% and 36 ± 18% in TE-FWD-ALT, for the first trials only and first and second trials together, respectively. When the feedback was reactivated, the performance recovered to a similar level as before, that is, 57 ± 13% (first trials) and 63 ± 11% (first and second trials) in TE-FDB-ALT, despite the fact that the system behavior was in this case significantly changed (Figures 8(a) and 8(b)). There is an indication that during the closed-loop control the subjects tended to make smaller force errors in the unsuccessful trials (Figure 8(c)); that is, in the failed trials in which object slipped from the grasp, the generated forces were closer to the target window if the subjects were provided with the electrotactile force feedback. However, the differences were statistically significant only between TE-FWD and TE-FDB-ALT. Finally, when the feedback was delivered, the subjects were more successful in using the outcome of the previous trial (slip or break) to modify the force and correctly grasp the same object from the second chance (83% and 64% versus 43% and 27% for the SR in corrections in Figure 8(d)). It can be noted from the standard deviations that the results were variable between the subjects. For example, the subject success rates (Figure 8(b)) in the two conditions with feedback (TE-FDB and TE-FDB-ALT) were in the ranges 55–90% and 55–85%, respectively. Without feedback, the performance could be as low as 20% in TE-FWD and 10% in TE-FWD-ALT, but it could also reach up to 60 and 65% (best results), respectively.

**Figure 9:**
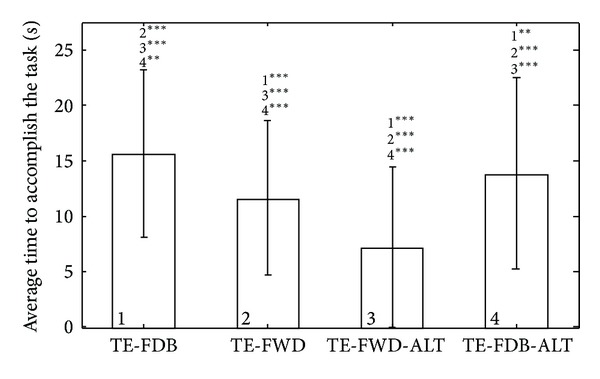
Average time to accomplish the task (ATA) (mean ± standard deviation) in different testing conditions. Providing feedback increased the time to accomplish the task.

The average time to accomplish the task (Figure 9) was significantly longer during closed-loop control conditions, that is, 16 ± 8 s and 14 ± 9 s for TE-FDB and TE-FDB-ALT versus 12 ± 7 s and 7 ± 7 s for TE-FWD and TE-FWD-ALT. There was a statistically significant difference also between the two conditions in which the feedback was provided (TE-FDB versus TE-FBD-ALT). This can be due to the fact that the altered system responded faster, resulting in shorter trials. Also with this system, the subjects were almost two times faster without feedback than with feedback, suggesting the lack of meaningful control in TE-FWD-ALT. Similar to the success rates, note that there was a large variability between the subjects also in the time that they used to accomplish the task.

**Figure 10:**
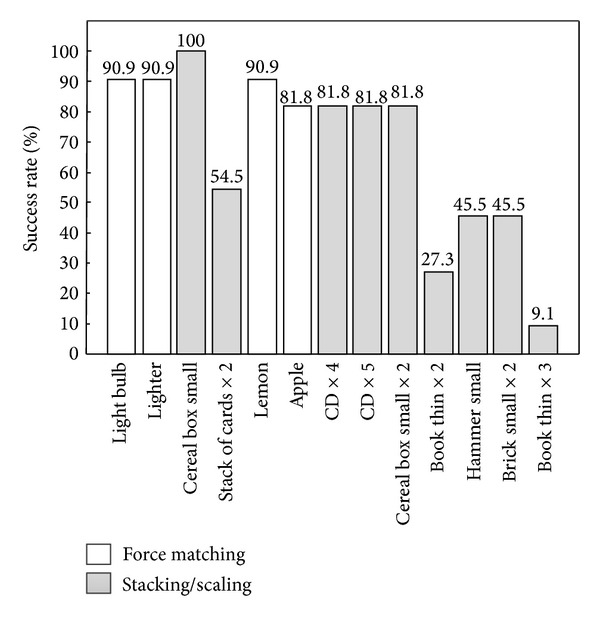
Success rates in generalizing closed-loop control from a reference object to a test object (see Table 2). The task was to grasp an object that was similar to the reference (white bars) or represented a stacked/scaled version of the reference (gray bars). The objects are ordered according to the weight (from lighter to heavier). For the small forces (lighter objects), the subjects were very successful in both tasks.

The results of TE-FDB-GEN test are given in Figure 10. The test evaluated the ability of the human subjects to generalize the closed-loop control to an object that was similar to a reference one (force matching task) or represented a stacked/scaled version of the reference (see Table 2). In the case of lighter objects, the subjects were very good in generating grasping forces for the similar test and reference objects and also in up-/downscaling of the force to reflect the up-/downscaling of the reference object (success rate > 80%, except for one outlier). However, when the force that the subjects had to generate increased (heavier objects), the success rate decreased to around 50% for the normalized forces of approximately 0.5 and to only 9% for the normalized forces of 0.8.

## 4. Discussion and Conclusions

In this study, we have developed a test system for the closed-loop control of force based on electrotactile feedback. The system operates in real time and integrates a setup for virtual grasping using a dynamic model of a single degree of freedom prosthetic hand. We have used this tool to investigate different properties of closed-loop force control during grasping of a set of daily life objects spanning a full range of normalized weights (0-1).

There are only a limited number of studies evaluating objectively the role and utility of closed-loop control in prosthetics, especially regarding the electrotactile feedback. Contrary to some other studies which showed no or limited improvement during closed-loop force control [11, 16, 17], the results in the current study are very optimistic with respect to the benefits of feedback.

After training shortly with only 5 objects (TR-ELE-VIS and TR-ELE-1) sampling the range of weights (from light to heavy), the subjects understood the “meaning” of electrotactile feedback and learned how to scale the force (tactile sensation) with the expected weight of the object. This first step (i.e., a basic introduction) facilitated the future training so that the subjects “learned” novel objects (TR-ELE-2, Figure 5) using only electrotactile feedback at the same pace as when they were learning objects assisted by both visual and electrotactile force feedback (TR-VIS-ELE and TR-ELE-1). Finally, after a short training which lasted less than 30 min in total, the subjects were able to grasp 10 objects of very different weights (from an egg to a hammer) with a success rate of 72% in total. As one of the future steps, it would be interesting to evaluate the effect of a more extensive training (longer time and/or more objects to grasp). As demonstrated in different context (i.e., object manipulation rather than grasping) and for vibrotactile feedback [24], the training is very important for performance, even more than the actual stimulation setup [25].

We have also shown that in this particular task the feedback was truly instrumental for good performance. The success rates dropped significantly with purely feedforward control (Figure 8), and also as demonstrated by the force profiles (Figure 7), the subjects used very different control strategies with and without the feedback. The subjects relied on the feedback both to adjust the force while grasping an object and also to correct the control based on the outcome of the previous trial. Both of these mechanisms were significantly less effective in the feedforward control. Finally, the closed-loop control showed to be very robust with respect to the change of system parameters. When the rate of change of force was doubled, the performance did not significantly change. There were no statistically significant differences in any of the outcome measures between TE-FDB and TE-FDB-ALT (Figure 8), except for the time to accomplish the task (Figure 9). The feedback improved the performance but at the expense of the longer time to accomplish the task due to a more complex processing that had to be accomplished by the subject. We have also demonstrated (Figure 10) that closed-loop control could be successfully used to grasp not only objects that were trained, but also novel objects that were “derived” from the latter ones in nontrivial ways (stacking up and scaling).

In some of the previous studies investigating the closed-loop control using vibrotactile stimulation, the ineffectiveness of the closed-loop control might be due to the actual experimental task. In some studies, only two [16] or three [10, 17] target force levels have been used. In Cipriani et al. [11], many objects were tested but the subjects were not instructed explicitly to follow the economical grasping paradigm. Saunders and Vijayakumar [16] used a binary switch as the controller and a single, constant rate of force increase/decrease. It might be that in these cases it was easier for the subjects to learn the task and system dynamics, after which they could “switch” to mostly feedforward control. In the current study, we have used more force levels and also an analog control interface (joystick) with continuous system dynamics (integrator). A greater variety and a relatively brief training could have made the feedback information essential for accomplishing the task. Note that this context is similar to the one that a user of a prosthesis will face in the real life (e.g., many different objects to handle). However, the reliance on feedback likely depends strongly on the provided training; it might be that, with a longer training, the subjects would eventually switch to feedforward control. An interesting outcome was that also in this study the subjects could reach a success rate of around 40% by relying purely on the feedforward control.

In the study by Meek et al. [13], the object to be grasped had a constant weight and the breaking force has been modified in different conditions. Similarly, Zafar and Van Doren [21] used a single target force level and several values for the width of the target force window. These studies have therefore shown that feedback improves the precision when reaching a single level of target force. In the current study, however, we have demonstrated that the feedback improved the performance when reaching a broad range of target force levels. In a recent conference paper by Witteveen et al. [15] a somewhat similar setup was used. However, vibrotactile stimulation was investigated and dynamic behavior of the prosthesis was not considered.

In general, the subjects were more successful when controlling lower forces, that is, when handling lighter objects. There might be two reasons for this. The first one is the linear scaling of the pulse width that we have used for information coding. It is well known that in general the intensity of the sensory stimulus does not map linearly to the intensity of perceived sensation [23]. For the higher pulses widths, larger absolute steps have to be made in the pulse width to produce a just noticeable difference in the elicited sensation [26]. We could have used a different scaling (e.g., power law or exponential function [27]). However, there is no agreement in the literature about the exact parameters of this mapping [8, 28], and the goal in this initial study was to test what can be done using the simplest approach. The second reason for the difficulties in handling heavy objects could be the habituation, which is more pronounced at higher intensities [7, 8]. While adjusting the force around the target window, the subjects might have lost the basic sensitivity in discriminating the changes in the intensity of the electrotactile stimulation. Importantly, most of the grasps in daily life are performed with light to medium objects, and also fine force control is mostly needed in the low to medium ranges, since the heavier objects are usually more robust. Nevertheless, the possibility of losing the basic sensitivity in the perception of electrotactile stimulation due to nonlinear psychometric function and/or habituation is an important problem. It affected the results in this study as described above and it therefore needs to be resolved for the future experiments, especially when considering the intended practical application. One possible approach could be to use the intermittent stimulation to decrease the habituation as successfully demonstrated in [29]. Moreover, in addition to the aforementioned scaling laws, different modulation schemes (e.g., simultaneous intensity and frequency modulation) could be tested to increase the discriminability of the stimulation [30].

Finally, in the next paragraphs, we point out certain limitations of the current study. It was not our goal to capture the full intricate complexity of the real life grasping task in which there are numerous factors affecting the selected grasping strategy, control, and progression. For example, grasping strongly depends on the object properties (e.g., texture and stiffness) and geometry, hand-object interaction (contact points), prosthesis features (e.g., nonideal dynamic response), and the functional goals (e.g., strong, stable grip versus fine manipulation). We implemented a virtual setup including certain realistic features (e.g., real time operation and set of real life objects) but we also assumed an ideal feedforward interface (joystick), prosthesis dynamic response (pure integrator), and contact dynamics (contact stability depended only on the grasping force). These simplifications were however intentional since the goal was to isolate a specific aspect that was of most interest in the current study: the general utility and characteristics of the electrotactile feedback during closed-loop control of grasping force. Importantly, some of the aforementioned, presently disregarded factors can be accommodated by our virtual grasping setup and investigated in the future experiments, as explained later.

The experimental protocol and the results of the current study can be discussed from the viewpoint of the common mechanisms of human perceptual and motor learning [31]. Namely, the research in this field has demonstrated that a rapid improvement in performance when an individual is first exposed to a novel task is a general characteristic of human learning both in motor and sensory domains. In the context of the current study, the success that the subjects have demonstrated during the tests evaluating the closed-loop control (TE-FDB, TE-FDB-ALT, and TE-FDB-GEN) could reflect this fast learning paradigm. Importantly, since the task at hand was closed-loop control, the learning took place in both domains simultaneously, integrating tactile feedback with motor commands (sensory-motor integration learning). However, it is still unclear if the achieved performance is just due to a normal, short term adaptation to the given sequence of motor tasks, or it reflects a more robust sensory-motor representation which could be stable over time (consolidated memory) or in different scenarios (randomized tests). This is an important question that needs to be addressed in the future studies by, for example, repeating the tests in several sessions over different days to assess the relevant mechanisms (e.g., stabilization, between-session and generalization of learning).

It is well known from the general psychometry [23] that sensory perception is affected by many internal and external factors (e.g., subject concentration) and that it can be therefore very variable both within session and between subjects. Since the performance in sensory processing is instrumental for the execution of the closed-loop control task, this could be a possible explanation for the variability of the results (i.e., large standard deviations in Figures 8 and 9). Also related to this, the number of subjects in this study was limited but sufficient to reach general conclusions (e.g., feedback versus feedforward) with statistical significance. However, a larger pool of subjects needs to be tested in order to assess with more confidence the actual baseline performance values in each of the tested conditions.

The setup developed in this study is very general and can be used to implement many different experimental scenarios for testing of the closed-loop control. It relies on modelling and virtual objects, which make it flexible, while at the same time it provides a realistic behaviour through real time performance. A more sophisticated and realistic model of a prosthesis can be easily implemented by changing few parameters of a Matlab Simulink block (e.g., using an integrator with a lag element and a pure time delay). Also, this is an ideal environment for testing how different feedback variables (position, velocity, force, and jerk) or modes of control (position or force control) affect the closed-loop performance. The developed test bench therefore provides a high flexibility in implementing real time closed-loop control scenarios that could generate important insights about the various aspects of artificial sensory feedback in prosthetics. Importantly, one should keep in mind that these experiments are still conducted in well-controlled conditions and by using an abstraction (model) of reality and that therefore the ultimate test of these results is an actual real life assessment. We intend to use the insights from this and similar virtual reality experiments as general guidelines for designing prototype systems, which will then be evaluated in subjects (healthy and amputees) operating real prosthesis to accomplish practical tasks (e.g., grasping real life objects).

## Figures and Tables

**Table 1:** Objects used in the tests.

Object number	Object	Normalized min/max force	Object number	Object	Normalized min/max force
1	Egg	0.05/0.2	12	Wine glass empty	0.15/0.3
2	CD	0.05/0.2	13	Wine glass half full	0.28/0.4
3	Orange	0.2/0.4	14	Light bulb	0.05/0.2
4	Wine glass full	0.35/0.5	15	Brick small	0.45/0.6
5	Bottle half full	0.5/0.7	16	Hammer small	0.6/0.8
6	Hammer	0.75/0.97	17	Lighter	0.05/0.2
7	Stack of cards	0.08/0.25	18	Lemon	0.2/0.4
8	Cereal box small	0.15/0.3	19	Apple	0.2/0.4
9	Cereal box	0.3/0.5			
10	Book thin	0.3/0.5			
11	Brick	0.75/0.9			

**Table 2:** Objects used in TE-FDB-GEN.

Reference object	Test object
Egg	Light bulb
Egg	Lighter
Orange	Apple
Orange	Lemon
Stack of cards	Stack of cards × 2
Cereal box	Cereal box small
Cereal box small	Cereal box small × 2
CD	CD × 5
CD	CD × 4
Thin book	Thin book × 3
Thin book	Thin book × 2
Small hammer	Small brick
